# Serial C-Reactive Protein Point-of-Care testing to optimize antibiotic treatment in hospitalized children with signs of infection in Zanzibar: A feasibility study

**DOI:** 10.1371/journal.pgph.0004777

**Published:** 2025-12-23

**Authors:** Daniel Joshua, Hanna Rahimi, Jeremiah Seni, Mayassa Ally, Muhiddin Omar, Jyoti Joshi, Farid Ali, Said Mohammed, Anja Poulsen, Stine Lund, ZAN-TOTO Collaborators

**Affiliations:** 1 Zanzibar Health Research Institute (ZAHRI), Unguja Island, Tanzania; 2 Global Health Unit, Department of Paediatrics and Adolescent Medicine, Copenhagen University Hospital Rigshospitalet, Copenhagen, Denmark; 3 Department of Microbiology and Immunology, Weill-Bugando School of Medicine, Catholic University of Health and Allied Sciences (CUHAS), Mwanza, Tanzania; 4 Department of Preventive Services and Health Education, Ministry of Health, Zanzibar, Tanzania; 5 The International Centre for Antimicrobial Resistance Solutions, Copenhagen, Denmark; 6 Public Health Laboratory Ivo de Carneri (PHL-IdC), Pemba Island, Tanzania; 7 Department of Neonatology, University Hospital of Northern Zealand, Hilleroed, Denmark; Child Health Research Foundation, BANGLADESH

## Abstract

Bacterial infections are among the leading causes of morbidity and mortality in children, especially in low- and middle- income countries. As a result, antibiotics are frequently prescribed to children with signs of infection, even when cause may not be bacterial. This contributes to antimicrobial resistance (AMR), a global health concern. Integrating serial CRP point-of-care testing with culture and susceptibility testing may improve decision-making by facilitating earlier differentiation between bacterial and non-bacterial infections, promoting more appropriate antibiotic use.The study examined the feasibility of serial CRP POCT to guide discontinuation of antibiotic treatment in children in selected hospitals in Zanzibar, and to determine barriers to methods and procedures for the upcoming randomised controlled trial (RCT) (ISRCTN25937092).This prospective, individually randomized feasibility study was conducted between February 5 and March 3 2024 in two hospitals in Zanzibar. Research assistants and healthcare workers (HCWs) were trained on the use and interpretation of CRP POCT. Eligible neonates and children were randomized to CRP-guided or standard care antibiotic management. Feasibility was assessed using Bowen’s framework; clinical outcomes were interpreted as exploratory.Eighty-two children participated, with a 96.0% follow-up rate. The CRP POCT intervention was rated “very important” by 89.7% of 58 HCWs. Adherence to CRP-based antibiotic guidance was high, though early discharges due to caregiver pressure were noted. The intervention integrated successfully into existing workflows and HCWs adapted the flowcharts in practice. Exploratory analysis showed CRP guidance reduced antibiotic treatment days in neonates with signs of infection (5.1 vs 6.6 days), and children aged 6 months to 12 years with febrile illness or diarrhoea (4.8 vs 6.7 days) compared to standard care, but the study was not powered for statistical inferences. This study suggests that the RCT of serial CRP POCT in guiding antibiotic treatment decision is feasible, and operationally implementable in this setting.

## Introduction

Infectious diseases are a global health problem, especially in children. Although major progress in combating infectious diseases has been made, the burden of these diseases in children under five years of age remains significant, particularly in low- and middle-income countries (LMICs), costing the lives of approximately 2.61 million children annually worldwide [1–4]. Reports from LMICs, including Tanzania, show that respiratory tract infections, diarrhoeal diseases and septicaemia predominate among children [4–6]. Furthermore, within the paediatric population, neonates remain the most affected group. About 2.3 million children die within the neonatal period every year, with the highest mortality rate in Sub-Saharan Africa with 27 neonatal deaths per 1,000 live births in 2022, and improving neonatal survival and health is still a global agenda [7,8]. One-third of all neonatal deaths worldwide are due to sepsis [9]. However, the incidence and case fatality rates are distributed unevenly, with noticeable differences within and across countries. The incidence of neonatal sepsis in high-income countries (HICs) ranges from 1 to 5 cases per 1000 live births, while this number is estimated to be 3.5-fold higher in low-income countries (LICs) [9–12]. Similarly, the case fatality rate in LMICs is seen to reach up to 65%, whereas it is less than 2% in HICs [13,14].

Antimicrobial resistance (AMR) has emerged as one of the leading global public health threats of the 21st century, and according to WHO and other groups and researchers, AMR requires a coordinated global action plan to address it [15–18]. Studies across the globe, including Zanzibar, are showing alarming rates of AMR in childhood infections [6,19–24]. The burden of AMR is driven by overarching societal and health care system factors [25]. Nevertheless, one of the main driving factors has largely remained to be the overuse and inappropriate use of antibiotics [18]. One multi-centre study in Tanzania mainland reported an overall antibiotic usage among 948 admitted patients to be 62.3%, predominantly among children and surgical patients, emphasizing the need for targeted interventions to the paediatric population [26]. Noncompliance with standard treatment guidelines as well as treatment without supportive laboratory results were some major contributing factors to irrational use of antibiotics underscoring urgent actions to prevent even higher morbidity and mortality from infectious diseases.

Symptoms such as fever and diarrhoea can result from a variety of different pathogens, which are difficult to distinguish from one another based on clinical presentations alone. In many low-resource settings including Zanzibar, this diagnostic uncertainty frequently leads to empirical antibiotic prescribing even in cases where bacterial infection is unlikely. For example, children with diarrhoea are commonly treated with antibiotics across Tanzania health facilities [27,28] and the pattern is driven by clinical caution and limited laboratory capacity. Therefore, one approach to reduce unnecessary antibiotic use is to support healthcare workers (HCWs) with diagnostic strategies that aid clinical decision-making by helping to distinguish patients likely to have bacterial infections requiring antibiotics from those with viral infections who may not benefit from them. C-reactive protein (CRP) is an acute phase reactant and inflammatory protein, often used as a fast and cost-effective marker of infection [29,30]. Serial CRP measurements that remain within the normal range have an almost 100% negative predictive value for bacterial infection and can therefore be safely used to support early discontinuation of unnecessary antibiotic therapy [31,32]. To our knowledge, there is no evidence on serial CRPs utility in hospitalized children with infections in LMICs. Decisions about antibiotic treatment are often complex at this level of care and are influenced by severity of disease, clinical diagnosis with limited laboratory diagnostics access, and requested tests often take long to process. Therefore, further studies in hospitalized children in low-resource settings, particularly with regards to feasibility and effects on antibiotic prescribing and cost-effectiveness of serial CRP testing, are needed.

This study aims to assess the feasibility of serial CRP Point of Care Test (POCT) guided antibiotic stewardship in neonates and children with suspected infection in hospitals in Zanzibar, and to determine possible barriers of the methods and procedures intended to be used in the upcoming randomised controlled clinical trial (RCT) (ISRCTN25937092) [33].

## Materials and methods

### Ethics statement

This study was approved by Zanzibar Health Research Ethics Committee (ZAHREC) (Ref. NO. ZAHREC/04/AMEND/DEC/2023/11). Parents or caregivers gave informed consent to participate in the study before taking part. The consent process involved providing detailed verbal and written information in Swahili language about the purpose, procedure, risks and benefits of the study, use of clinical data and performance of follow-up. Parents and caregivers were given the opportunity to ask questions and only those who voluntarily agreed signed the consent form. Consent was documented by both parent or caregiver’s signature or thumbprint and the signature of a trained research assistant. The research team were directly in contact with the health care providers (including all attending doctors), and throughout the study, patients’ interests were upheld to ensure favourable management outcomes.

### Study design and setting

The study was a prospective individually randomised feasibility study designed in preparation for the Zan-toto Randomized Controlled Trial in Zanzibar, Tanzania. To determine possible barriers of the methods and procedures intended to be used in the upcoming RCT, feasibility of using the CRP POCT was assessed using the framework provided by Bowen et al whereby mixed qualitative and quantitative mixed-methodology is used describing acceptability, demand, implementation, practicality, adaptation, integration, expansion, limited-efficacy testing (Table 1) [34]. Limited-efficacy testing followed the planned methodology of a prospective, multicenter, individual randomised controlled trial (RCT) with 28 days follow-up conducted in Vitongoji Hospital (VH) and Mnazi Mmoja Hospital (MMH).

**Table 1:** Bowens feasibility framework (adapted).

Area of focus	Definitions according to *Bowen et al*	Procedures in current study
1. Acceptability	• To what extent will HCWs accept the new idea?	• Questionnaire assessing acceptability of CRP POCT among HCWs
2. Demand	• Is there a demand? • Is it fit within the organisational culture?	• Assessing current use of antibiotics in the country (existing literature) • Questionnaire assessing demand of CRP POCT among HCWs • Exploratory meetings with employees from the Ministry of Health
3. Implementation	• Can the new idea be successfully implemented?	• Assessment of implementation done by site supervisors throughout the study period
4. Practicality	• Implementation with existing means, resources, and circumstances?	• Assessment of practicality within current context done by site supervisors throughout the study period
5. Adaption	• To what extent can a new idea perform when changes are made for a new format? • Degree to which similar outcomes are obtained in a new format?	• Assessment of adaption done by site supervisors throughout the study period
6. Integration	• To what extent can it be integrated into the existing system?	• Assessment of integration done by site supervisors throughout the study period
7. Expansion	• To what extent can the method be expanded?	• Assessment of expansion done by site supervisors throughout the study period
8. Limited-efficacy testing	• Does the new idea show promise of being successful in the intended populations? • Intended effects on key intermediate variables	• Performance of descriptive analyses as well as Mann-Whitney and two-sample t-test on the study data

### Study population

Neonates at the maternity ward and admitted neonates and children at the paediatric wards were screened for eligibility, in the four-week period from February 5^th^ to March 3^rd^ 2024.

### Inclusion criteria

- Subgroup 1: Well-appearing newborns at birth with gestational age ≥37 weeks or birthweight ≥2500 grams, and risk factors of early onset sepsis (EOS) according to the WHO criteria
  - Rupture of membranes over 18 hours before delivery and/or
  - Maternal fever (over 38 °C) during labour and/or
  - Amniotic fluid foul smelling or purulent
- Subgroup 2: Neonates between 0–28 days old, gestational age ≥ 37 weeks or birthweight ≥2500 grams, admitted with suspected infection (clinical signs judged by the clinician)
- Subgroup 3: Children between 6 months to 12 years old admitted with
  - Febrile illness with temperature above 38 °C or below 36 °C at admission, or a history of febrile illness within the last 72 hours OR
  - Diarrheal disease (defined as the passage of 3 or more loose or liquid stools per day) with or without fever

### Exclusion criteria

- Severely ill, defined as neonate or children requiring urgent life-saving interventions such as resuscitation or immediate administration of antibiotics where measurement of CRP POCT would delay the treatment process
- Neonates with major congenital malformations that require hospital admission
- Patients with known immunosuppression or severe chronic disease (human immunodeficiency virus (HIV), hepatic disease, history of neoplastic disease, long-term systemic steroid use, severe acute malnutrition (SAM) or similar conditions as assessed by the HCW)
• Positive rapid diagnostic test for malaria – These children were excluded because malaria is typically treated with antimalarials rather than antibiotics and elevated CRP levels due to malaria could confound the evaluation of CRP-guided antibiotic decision-making
- Neonate/child has been given antibiotics within 24 hours before admittance
- Parents/caregivers are unable or unwilling to provide informed consent
- Parents/caregivers are unable or unwilling to participate in follow-up procedures

### Study outcomes

The primary aim of this study was to assess the feasibility of implementing CRP POCT to guide antibiotic treatment in neonates and children with suspected infection. Feasibility was evaluated using Bowen et al.’s framework which includes eight domains, acceptability, demand, implementation, practicality, adaptation, integration, expansion and limited efficacy testing. Other feasibility outcome assessed included adherence to CRP-guided.

Additionally, exploratory clinical outcomes were collected to inform the design of the future randomized controlled trial. These included duration of antibiotic treatment, length of hospital stay, re-admission within 28 days and mortality.

### Study procedures

Neonates and children meeting the inclusion criteria were enrolled during a predefined four-week recruitment period, as agreed upon by the study team, to ensure sufficient lead time for the initiation of the definitive randomized controlled trial. Written informed consent was obtained from the parent or caregiver of each eligible child prior to enrollment in the study, and then, the child was randomly assigned to either CRP-guided or standard-of-care antibiotic treatment. The Aidian QuickRead go CRP POCT machines (Aidian, Espoo, Finland), and necessary equipment were supplied at VH and MMH, and research assistants (RAs) as well as HCWs received training about the study and in the use and interpretation of CRP POCT supporting the clinical evaluation of the neonate and child.

For the intervention group, CRP POCT was measured at 18–24 hours of life for neonates at risk of EOS (these are well-appearing newborns with risk factors who have not yet shown any symptoms), or for neonates with signs of infection and children with febrile illness or diarrhoea at admittance and again after 18–24 hours of treatment. Antibiotic treatment in the intervention group was guided by the CRP results with flowcharts supporting the clinician’s decision of treatment including discontinuation of antibiotic treatment when repeated CRP results did not indicate bacterial infection.

Based on the bacterial infection - clinical risk profile of each subgroup, CRP cur-offs were selected to reflect differing pre-test probabilities and baseline inflammatory responses across the three subgroups. A higher threshold was applied in well-appearing neonates to preserve specificity and reduce unnecessary treatment, while lower thresholds were used in symptomatic neonates and older children to prioritize sensitivity and patient safety.

For neonates in subgroup 1 randomized to intervention, a CRP measurement taken within the first 18 – 24 hours of life was used to guide antibiotic decision. A measurement of <30mg/l indicated that the neonate most likely did not need antibiotics [31,35] and the parents and the parents were informed of seeking care if the child indicated any danger signs. CRP levels 30mg/l – 50mg/l warranted a caution and the neonate was admitted, clinically assessed with a repeat CRP measurement after 6 hours. CRP measurement >50mg/l indicated that neonate required antibiotic treatment for a possible EOS. When the neonate at any time showed signs of neonatal infection, antibiotic treatment was started according to the Zanzibar standard treatment guidelines.

For neonates in subgroup 2 randomized to intervention, CRP levels, measured at admittance and after 18–24 hours of treatment, if both CRP values were below 10mg/l, antibiotics are most likely not needed [36–38]. CRP levels above 100mg/l suggested severe disease and antibiotics were started and continued promptly.

**Fig 1:**
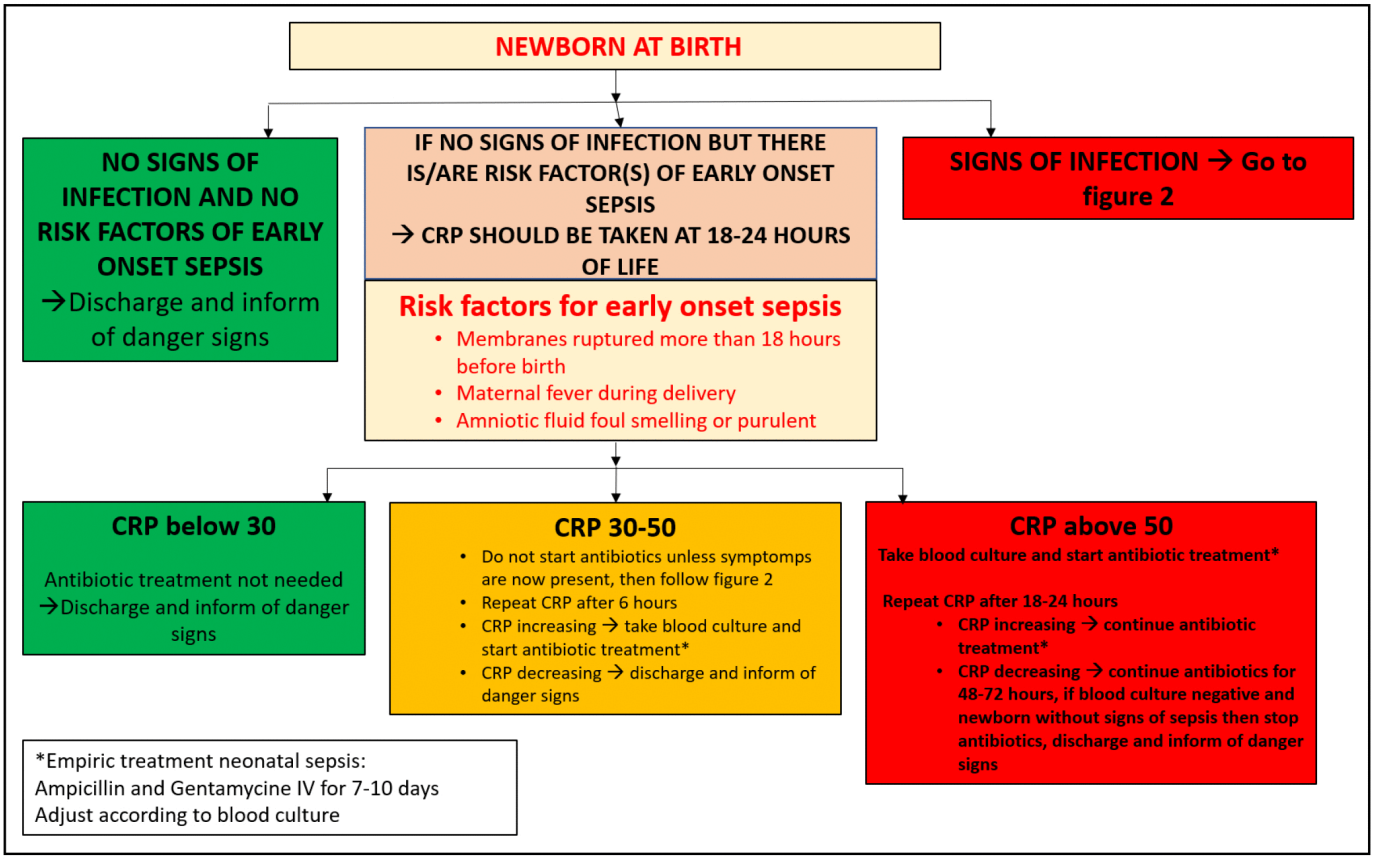
CRP intervention flowchart for neonates with risk factors of early onset sepsis (EOS).

**Fig 2:**
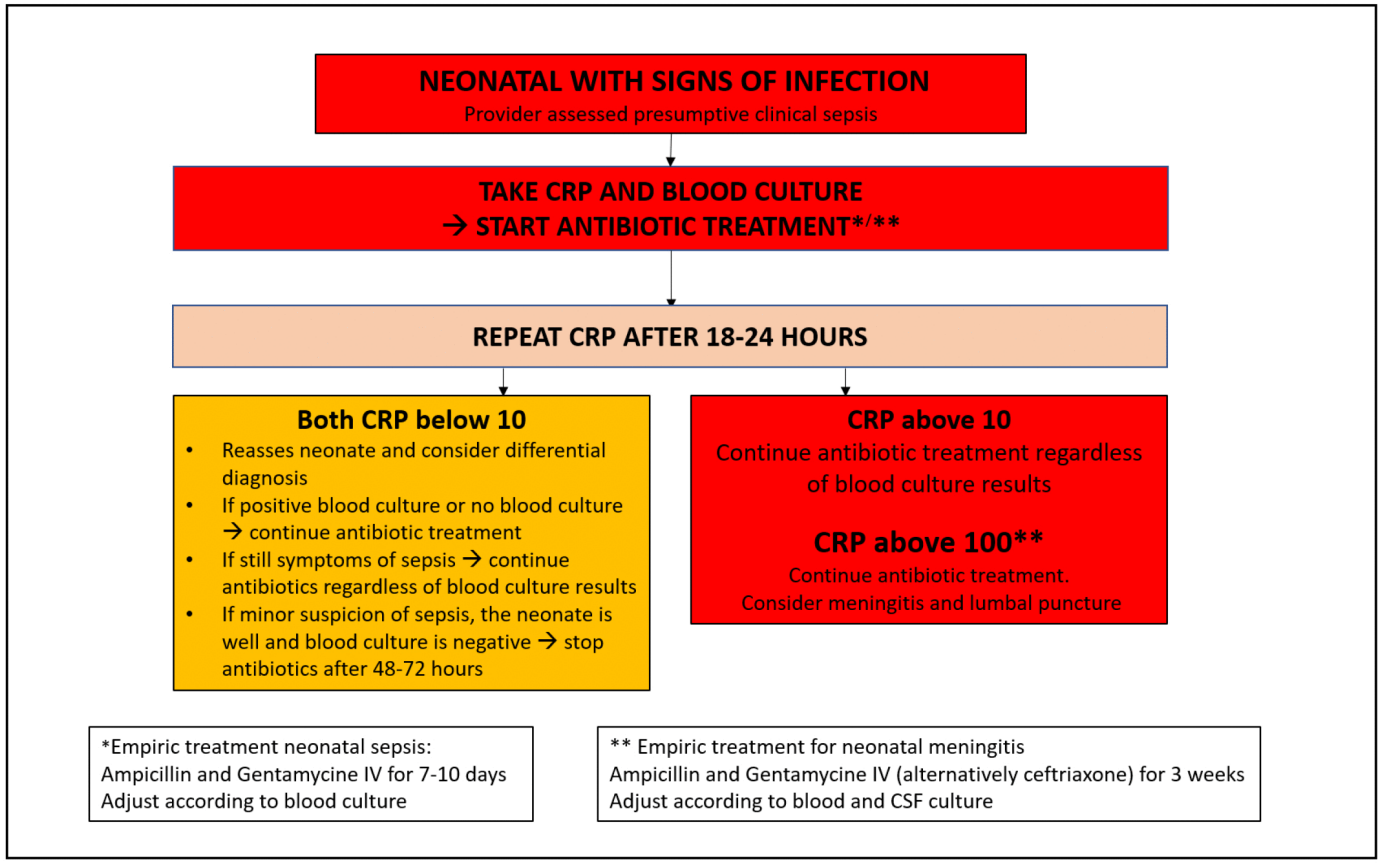
CRP intervention flowchart for neonates with signs of infection.

**Fig 3:**
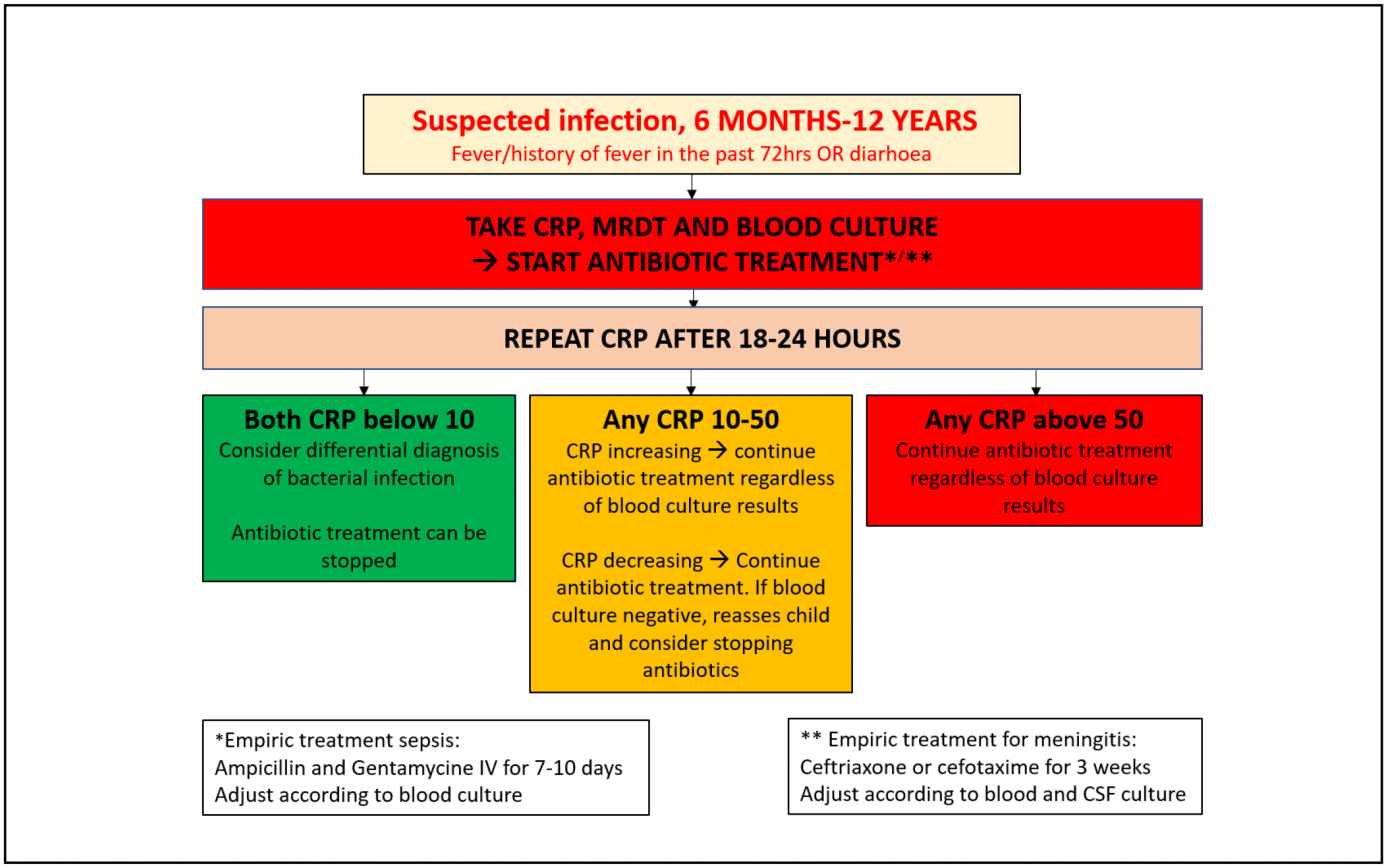
CRP intervention flowchart for children with febrile illness or diarrhoea.

Children in subgroup 3 randomized to intervention, CRP levels, measured at admittance and after 18–24 hours of treatment, if both CRP values were below 10mg/l, the disease was considered likely not severe and antibiotics are most likely not needed, can either be discontinued or not be started. If second CRP levels 10mg/l – 50mg/l, antibiotics might be considered depending on the trend of CRP levels. CRP readings >50mg/l suggested that antibiotics were likely needed and treatment to be continued [39–41] (Figs 1–3).

At all times, the HCWs were instructed to take the clinical picture into account together with the value of the test. A system for collection of blood cultures was established during the feasibility study period, but due to some delay in the startup process and quality assurance procedures, we decided not to report on the culture results in this study.

In the control group, usual standard-of-care antibiotic treatment was given. In both groups, follow-up was carried out through phone calls to the caregivers on day 7 and day 28 after discharge in order to record clinical outcome and/or provide more clinical support to children wherever needed.

RAs were responsible for carrying out and documenting data from inclusion and eligibility assessment, enrollment, randomization, informed consent, CRP POCT and structured questionnaires during admission as well as at discharge and follow-up. Site supervisors oversaw the data collection and ensured accuracy of collected data. RAs had excellent skills in Swahili and were intermediate in English. All research data was entered electronically directly on tablets into REDCap.

Both intervention and control group had a background questionnaire about demographics, socio-economics and health seeking behaviour. Furthermore, participants were followed daily with a clinical registry form, where information about clinical assessment, diagnosis, and hospital management including antibiotic use was recorded. At the time of discharge, referral or death, a discharge form was filled. Moreover, the parents or caregivers were contacted at day 7 and 28 after discharge and asked about the health status of the child. Follow-up questionnaires included questions on recovery, symptoms, antibiotic use as well as complications or hospitalisations. Lastly, as part of the assessment of the feasibility, HCWs were asked to answer a questionnaire on their professional education, work experience as well as acceptability of using CRP.

The RCT design of the limited-efficacy testing and the questionnaires on acceptability and demand involved quantitative analysis, but in order to assess the implementation, practicality, adaption, integration and expansion, observations and continuous discussions between site-supervisors and employees from the Ministry of Health took place, which explored perceptions, provided context for understanding practical and logistical challenges of the intervention, and determined barriers to of the methods and procedures.

### Randomization and blinding

Individual randomization was carried out by the responsible RA directly in REDCap. Participants were randomized (1:1) to either management according to the CRP intervention or standard care. Randomization was stratified within the three sub groups. The responsible RA informed the HCW of the result of the randomization. Due to the nature of the study, blinding the clinicians and RA was not possible.

### Data analysis

Given the feasibility nature of this study, the primary aim was to descriptively assess feasibility outcomes based on Bowen et al.’s eight domain framework. Quantitative data were summarized using descriptive statistics including frequencies, proportions, means and median; and were further stratified by study groups (intervention vs. control). Descriptive analysis was conducted for; feasibility indicators including HCWs acceptability, adherence to CRP guidance, follow-up completion rates and enrollment outcomes (enrollment rate and exclusion reasons) also for exploratory clinical outcomes including duration of antibiotic treatment, length of hospital stay, readmissions and mortality.

Although the study was not powered to detect statistically significance difference, exploratory statistical comparison were conducted to inform the design of the upcoming trial. The continuous variables, antibiotic duration and hospital admission duration were assessed for normality using the Shapiro-Wilk test. Based on the normality assessment, the Wilcoxon rank-sum (Mann-Whitney) test was performed to compare the duration of antibiotic treatment between groups in subgroup 1 while the two-sample t-test was used for subgroups 2 and 3. Comparison of hospital admission duration intervention and control groups were performed using Wilcoxon rank-sum test across all subgroups due to non-normal distribution. A significance level of p < 0.05 was considered for all exploratory comparisons. All statistical analyses were conducted using Stata 15 software (StataCorp. 2017. Stata Statistical Software: Release 15. College Station, TX: StataCorp LP).

The evaluation of the dimensions of implementation, practicality, adaptation, integration, and expansion was informed through continuous, informal feedback mechanisms. Site supervisors and HCWs from the Ministry of Health engaged in iterative discussions and observations throughout the study period. These informal interactions provided real-time insights into HCWs and participant experiences on the practicality, logistical challenges, and procedural barriers. While these discussions and observations were not systematically documented, the feedback informed ongoing refinements to the intervention and study procedures. Key changes and adaptations were recorded in field notes and study logs maintained by the site supervisors to capture the evolution of the study implementation over time. Qualitative insights gathered through this iterative feedback process were thematically summarized based on the recorded changes and the research team’s reflections. This approach allowed us to identify recurring themes, practical barriers, and facilitators related to the intervention’s feasibility. This informal nature of the feedback mechanism provided valuable insights for real-time decision-making, and was therefore prioritized over systematic documentation (e.g., transcripts or structured notes) leading to a detailed qualitative analysis. This limitation was acknowledged and considered in the interpretation of findings.

## Results

### Feasibility outcomes

#### Participants recruitment and enrolment.

**Fig 4:**
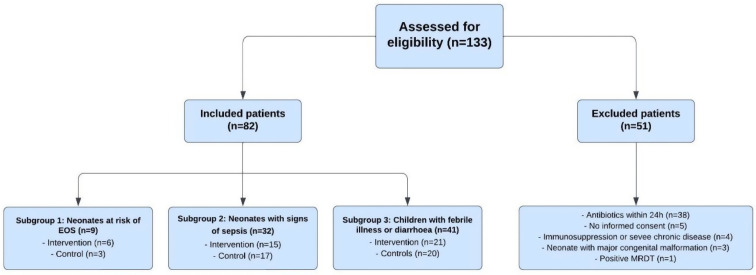
Flow chart of the study population.

A total of 133 neonates and children were eligible for inclusion in the four-week period, of which 82 neonates and children were enrolled in the study. A total of 40 participants were enrolled to CRP-guided intervention group and 42 to the standard care group. Baseline characteristics of the included participants, clinical symptoms and CRP values are presented in Table 2. Fifty-one neonates and children had one or more exclusion criteria, with “has been given antibiotics within 24 hours before admittance” being the most common reason (n = 38), followed by “parent/caregiver unable or unwilling to provide informed consent” (n = 5), “patient known with immunosuppression or severe chronic disease” (n = 4), “neonate with major congenital malformation that will require hospital administration” (n = 3), and “positive rapid diagnostic test for malaria” (n = 1) (Fig 4).

**Table 2:** Baseline characteristics, symptoms and CRP values of patients enrolled.

Variable	Intervention	Control
*Total number of participants, N (%)	42 (51.2)	40 (48.8)
Subgroup 1	6 (66.7)	3 (33.3)
Subgroup 2	15 (46.9)	17 (53.1)
Subgroup 3	21 (51.2)	20 (48.9)
**Age at admission, median (IQR)**		
Subgroup 1, days	0 (0 – 0)	0 (0 – 0)
Subgroup 2, days	3 (1- 9)	1 (0 - 3)
Subgroup 3, months	16.8 (10 - 24)	12 (12 - 18)
**Sex, n (n/N%)		
Subgroup 1, girls	4 (66.7)	2 (66.7)
Subgroup 2, girls	10 (66.7)	13 (76.5)
Subgroup 3, girls	11 (52.4)	5 (25.0)
**Most common clinical symptoms at admission, n (n/N%)**		
***Subgroup 2***		
Fever	13 (86.7)	15 (88.2)
Tachypnoea	4 (26.7)	1 (5.9)
Feeding intolerance	3 (20.0)	1 (5.9)
Jaundice	6 (40.0)	9 (52.9)
***Subgroup 3***		
Fever	21 (100.0)	20 (100.0)
Cough	12 (57.1)	14 (70.0)
Diarrhoea	5 (23.8)	8 (40.0)
**First CRP value, n (n/N%)**		
***Subgroup 1***		
<10 mg/L	3 (50.0)	–
10 to 30 mg/L	0 (0.0)	–
30 to 50 mg/L	2 (33.3)	–
>50 mg/L	1 (16.7)	–
***Subgroup 2***		
<10 mg/L	11 (73.3)	–
10 to 50 mg/L	4 (26.7)	–
>50 mg/L	0 (0.0)	–
***Subgroup 3***		
<10 mg/L	11 (52.4)	–
10 to 50 mg/L	7 (33.3)	–
>50 mg/L	3 (14.3)	–
**Second CRP value, n (n/N%)**		
***Subgroup 1***		
<10 mg/L	0 (0)	–
10 to 30 mg/L	1 (16.7)	–
30 to 50 mg/L	1 (16.7)	–
>50 mg/L	0 (0)	–
2^nd^ CRP not applicable due to low 1^st^ CRP	3 (50.0)	
Missing value	1 (16.7)	
***Subgroup 2***		
<10 mg/L	9 (60)	–
10 to 50 mg/L	4 (26.7)	–
>50 mg/L	2 (13.3)	–
***Subgroup 3***		
<10 mg/L	11 (52.4)	–
10 to 50 mg/L	7 (33.3)	–
>50 mg/L	2 (9.5)	–
Missing value	1 (4.8)	–

*Subgroup 1) neonates at risk of EOS, 2) neonates with signs of infection, 3) children with febrile illness or diarrhoea.

**Percentages are calculated using the total number of participants within each subgroup and arm as the denominator.

#### Acceptability and demand.

Fifty-eight HCWs from the paediatric departments of the two participating hospitals completed the post-intervention questionnaire (S1 Text). This represented the total number of HCWs available during the study period. Of these, fifty-two (89.7%) HCW’s found CRP POCT to be “very important” for guiding antibiotic treatment in neonates and children with signs of infection and five (8.6%) found it to be “somewhat important”. Only 1 (1.7%) HCW did not find it important. Similarly, 49 (84.5%) HCWs believed CRP POCT could “significantly” help reducing unnecessary antibiotic prescription, and 9 (15.5%) HCWs answered “yes, to some extent”. Furthermore, 50 (86.2%) HCWs felt confident in interpreting CRP POCT results after the feasibility study.

#### Implementation, adherence and protocol deviations.

CRP testing was integrated into clinical workflows with high adherence to the intervention flowcharts. However, eight neonates and children (19%) in the intervention group were discharged within less than a week, some with and some without oral antibiotics, even though they had CRP levels that required 7 full days of IV antibiotic treatment. Some of the main reasons were high bed occupancy and caregiver preference for discharge.

#### Practicality and integration.

The CRP POCT was possible in the context. Staff adapted easily to the procedure and the presence of stable power supply and consistent staffing at both hospitals facilitated implementation. Data collection was through REDCap module on tablets and was completed reliably.

#### Follow-up and retention.

The follow-up rate in this study was 96% (76/79); 89% (8/9) in subgroup 1, 100% (29/29) in subgroup 2 and 95% (39/41) in subgroup 3. Most lost to follow-up was due to leaving the hospital without notice. Follow-up was achieved through phone contact at day 7 and day 28 post-discharge.

#### Adaptation and expansion.

Based on operation insights, adjustments were proposed for the main RCT and these included, minimum birth weight of included neonates adjusted to 2 kg, expansion to additional hospitals and improving follow-up tracking by collecting multiple phone contacts (at least 2) at admission.

A summary of feasibility outcomes categorized by Bowen’s eight-domain framework as illustrated in Table 3.

**Table 3:** Bowens feasibility framework (adapted) with outcomes of interest.

Area of focus	Outcomes of interest from the current feasibility study
1. Acceptability	• CRP POCT was acceptable for all HCWs
2. Demand	• There was a perceived demand for guidance in antibiotic use in childhood infections • There was an established need of guidance from paraclinical tests to improve decision making on antibiotic treatment in neonates and children • CRP POCT was found appropriate within the organisational culture
3. Implementation	• CRP POCT could be implemented • The study teams were able to carry out and implement the study at the hospitals
4. Practicality	• CRP POCT was possible in the context • The power source was stable • We became aware of challenges such as high bed occupancy rate and wish for discharge by caregiver, resulting in earlier discharge. • Protocol was adapted and phone numbers will be taken at admission in main trail
5. Adaption	• CRP POCT was possible when neonate at risk of EOS stays in maternity ward instead of being admitted to paediatric ward • Blood for CRP POCT was collected in different ways (capillary or venous), separately or while performing other procedures (IV-line insertion, blood sample or blood culture collection) • CRP POCT can be implemented in different patient groups without effect on clinical work other than antibiotic management
6. Integration	• CRP POCT fit within existing infrastructure • CRP POCT work in a local context with no disturbances on workflow
7. Expansion	• Expansion is possible with all practical factors considered • Protocol was adapted to include neonates from 2 kg in main trail • CRP POCT can be scaled up in more facilities
8. Limited-efficacy testing	• There was an added value of CRP POCT to guide on the appropriateness of antibiotic treatment • A small-scale reduction in antibiotic use duration was seen in CRP POCT intervention group, without compromising health outcomes (not powered for significance)

### Exploratory clinical outcomes

#### Limited-efficacy of the intervention.

Of the 82 enrolled participants, nine neonates were at risk of EOS (subgroup 1), 32 neonates had signs of infection (subgroup 2) and 41 children had febrile illness or diarrhoea (subgroup 3). The most common clinical symptoms at admission in the neonates and children with signs of infection was fever (28/32 in subgroup 2 and 41/41 in subgroup 3). For neonates in subgroup 2, other common symptoms were tachypnoea (5/32) feeding intolerance (4/32) and jaundice (15/32), while the children in subgroup 3 had cough (26/41) and diarrhoea (13/41). Most children had low first CRP levels <10 mg/L (25/42), and only one (17%) in subgroup 1 and three (14%) in subgroup 3 had CRP levels >50 mg/L. The second CRPs were likewise low; almost half of patients had CRP levels <10mg/L (20/42), and only two (13%) in subgroup 2 and two (10%) in subgroup 3 had CRP levels >50 mg/L. In subgroup 1, no patients had an increasing CRP. In subgroup 2, three patients (20%) had an increasing CRP, but only one (7%) had a second CRP level >50 mg/L. In subgroup 3, three patients (14%) had an increasing CRP, but all of them remained <50 mg/L.

The CRP guidance reduced the median number of days with antibiotic treatment in subgroup 2 (6 days in intervention group vs 7 days in control group), p = 0.22 and subgroup 3 (6 days in intervention group vs 7.5 in control group), p = 0.08. In subgroup 1, the median number of days with antibiotics was 0 in both intervention group and control group, p = 0.72 (Table 4). Re-admissions within 28 days were not observed in any participant across all study groups.

**Table 4:** Primary and secondary outcomes.

Variable	Intervention	Control	P-value
***Primary Outcome***			
**Antibiotic duration, median days (IQR)**			
Subgroup 1	0 (0-0)	0 (0-1)	0.72
Subgroup 2	6(2-7)	7(6-8)	0.22
Subgroup 3	6 (1 – 7)	7.5(5.5-9)	0.08
***Secondary Outcomes***			
**Hospital admission duration, median days (IQR)**			
Subgroup 1	1 (1 – 1)	2 (0 -2)	0.68
Subgroup 2	3 (2-5)	3(2-5)	0.61
Subgroup 3	3 (2-5)	3 (1.5-5)	0.53
**Mortality, n (n/N%)**			
Subgroup 1	0 (0)	0 (0)	
Subgroup 2	3 (20)	0 (0)	
Subgroup 3	0 (0)	0 (0)	

In subgroup 2, there were 9% (3/32) mortality cases. All three were in the intervention group and promptly started on antibiotics at admittance and remained under treatment until they deceased. The first case was a 1-day old baby that was admitted after birth with meconium aspiration, had a 2^nd^ CRP over 50 mg/L and died within 24 hours of admission. The second case was a 28-day old baby that had been admitted for 10 days, the case notes indicated aspiration pneumonia and herbal intoxication with both CRPs over 50 mg/L. The third case was a 15-day old baby with both CRPs < 10, with suspected aspiration pneumonia after having received oral rehydration solution for gastroenteritis. In subgroup 3, one child in intervention group was still symptomatic at follow-up day 7 but had recovered well at day 28. In the control group, two children had fever again at follow-up day 28 but were not re-admitted to the hospital.

## Discussion

This study demonstrated that it was feasible to use serial CRP POCT guided antibiotic stewardship in a low resource hospital setting. The vast majority of HCWs found CRP POCT to be an important and acceptable tool for guiding antibiotic use in neonates and children. Furthermore, all HCWs believed that CRP POCT could help reducing unnecessary antibiotic prescription in childhood infections, and most HCWs felt confident in interpreting the results after the feasibility study. Implementing and integrating CRP POCT in hospital services was practically possible in this context. The limited-efficacy testing indicated that CRP POCT has the potential to reduce antibiotic use with no evident safety concerns, although these exploratory findings should be interpreted cautiously.

Our findings are in consistent with previous studies from primary health care in a high-resource setting, where HCWs supported the introduction of CRP POCT into routine clinical practice to guide proper use of antibiotics in childhood infections and to reduce the use of antibiotics when repeatedly low CRPs levels are seen [42].

We identified a demand for diagnostic tests that support and improve antibiotic treatment decision for childhood infections in hospitals in Zanzibar. Our findings indicate that CRP POCT can be implemented and integrated in a both practical and adaptable manner in the daily clinical work. Overall, HCWs adhered to the flowcharts for the intervention group. However, we found that the frequently high bed occupancy ratio in the current context sometimes challenged the use of the CRP intervention flowcharts, and some patients had to be discharged early. Furthermore, a few parents pleaded early discharge due to socioeconomic factors that made it difficult for them to have their child admitted at hospital for a long time.

Data collection and data entry in REDCap worked well in the feasibility study, supporting efficient and reliable data management. Three caregivers provided no/incorrect/no longer valid phone numbers, leading to loss to follow-up. Therefore, all the phone numbers of the households will be collected at the first day of admission in the future main trial. Despite this minor loss, the high follow-up rate observed is encouraging, especially considering the use of telephone-based follow-up, which proved efficient and feasible in this low-resource setting [43,44].

Due to a lower-than-expected number of study participants in the feasibility study, we decided to include two more hospitals for the main trial as well as include neonates down to 2 kg, in line with evidence highlighting the vulnerability and underrepresentation of low-birthweight infants in LMICs neonatal trials [45]. Moreover, the procedure for identifying well-appearing neonates at risk of infection was improved, as we found the number of neonates with risk factors to be surprisingly low likely due to missed identification at birth. We initiated active postnatal screening of mothers in the maternity ward by RAs and maternity nurse for known risk factors such as prolonged rupture of membranes, maternal fever during labour or foul-smelling amniotic fluid. This allowed earlier recognition and inclusion of eligible neonates in the feasibility study, a strategy that will be incorporated in the main trial [46].

In the present study we were not able to report the blood culture results, although a system for collection was initiated. Delays in startup logistics and quality assurance prevented reliable processing. This highlighted an important gap in diagnostic infrastructure which will be rectified in the main trial where an improved setup and effective coordination with laboratory services to support timely blood culture diagnostics as part of the interventional arm clinical assessment will be established.

We were able to enroll 62% of eligible patients. As expected, the most common reason for exclusion was antibiotic use within 24h prior to study inclusion (75%). This emphasizes the high use of antibiotics in the present context, as previously reported in six hospitals in the Tanzania mainland and predominantly among children [26]. CRP POCT offers a potential solution for fast and cost-effective diagnosis, and the results from this study suggest that CRP POCT might be effective in guiding clinicians at hospitals in Zanzibar to use antibiotics more appropriate in childhood infections. Generally, a shorter duration of antibiotic use was observed in this study in neonates and children with signs of infection in the intervention groups, compared to controls. Given the small sample size of this feasibility study, the results should be interpreted with caution. Nevertheless, the findings are in line with a systemic review and metanalysis from 2018, that found that CRP-based algorithms could shorten antibiotic treatment duration in neonates with suspected infection [32].

Notably, neonates at risk of infection in control group did not receive prophylactic antibiotics according to WHOs guidelines, and a reduction in antibiotic duration was therefore not seen in the intervention group. These findings are supported by a prospective observational cohort study conducted in a hospital in Pemba Island of Zanzibar in 2022 [47], that found adherence to WHO guidelines for prophylactic antibiotic treatment to prevent neonatal infections to be low. Only 10.3% of neonates with risk factors for infection according to WHO guidelines received prophylactic antibiotic treatment, and only 3.4% received the correct antibiotic drug recommended in guidelines [48]. Our main trial will determine if the lack of prophylactic antibiotic use in neonates with risk factors for infection is consistent across hospitals in Zanzibar.

The implementation of CRP POCT was facilitated by the consistent clinical presentations observed among enrolled participants. Fever was the most common symptom among neonates and children with signs of infection, while cough and diarrhoea were commonly reported among children aged 6 months to 12 years. These symptoms are often non-specific and can be associated with both viral and bacterial aetiologies, making clinical differentiation challenging. Local studies from Tanzania have reported similarly high rates of empirical antibiotics use in children with similar symptoms due to diagnostic uncertainty [27,49]. The availability of CRP POCT provided HCEs with additional information to support antibiotic decision-making and this reinforces the practical utility of CRP POCT as a tool that integrates well within routine workflows where diagnostic capacity is limited.

There were three neonatal deaths in this feasibility study, all of which were showing possible signs of infection. Although these cases were part of the intervention group, the external Serious Adverse Event committee interpreted from the mortality reviews that the deaths were not to be related to the CRP intervention. All three neonates were severely symptomatic, all received antibiotics throughout their admission and died due to their severe clinical conditions combined with the hospital’s limited resources for managing critically ill patients. All remaining participants in this study, including those in the intervention group, recovered well during admission and were still doing well at the 7th and 28th day follow-ups.

The low levels of CRP measured in majority of participants in all intervention subgroups support the notion that most cases were likely viral or self-limiting bacterial infections. These data suggest that it is difficult for HCWs to differentiate between aetiologies of childhood infections, and that they are not completely sure about when to stop antibiotics in neonates and children with infections. This highlights the need for more education as well as practical tools, such as serial CRP POCT, to assist in a more rational antibiotic use in childhood infections.

This present study had limitations inherent to feasibility designs, nevertheless, the findings have directly informed the planning of the upcoming full randomized controlled trial (RCT). The sample size was relatively small and the study was not powered to detect statistically significant differences; the main trial will be adequately powered with a larger, multi-site sample to evaluate clinical outcomes. Short recruitment time which likely constrained recruitment; the RCT will be implemented for 12 months to enhance participants enrolment. Further, the small sample size and short recruitment period likely contributed to some imbalances in demographic and clinical characteristics between the groups as randomization may not have evenly distributed these characteristics. In the full-scale trial, a larger sample size will be considered to address this limitation and enhance balance between groups. External validity may be limited by the inclusion of only two hospitals but the inclusion of more varied facilities in the full trial will improve generalizability. Finally, the feasibility domains were assessed using descriptive methods; incorporating structured mixed methods approaches in future feasibility studies will provide more insight into implementation processes and acceptability [50].

## Conclusion

This study showed that serial CRP POCT in clinical decision-making on antibiotic treatment for children was feasible in a low resource setting. Serial CRP POCT has the potential to reduce antibiotic duration with no evident safety concerns, and this will be investigated in the upcoming larger RCT. Here, additional study sites will be included, and a birth weight down to 2 kg will be accepted for neonates. Lastly, efforts will be made to collect phone numbers from all households to improve follow-up data.

## Supporting information

S1 Text Healthcare workers post-intervention questionnaire. (PDF)

S2 Text Sub-study 2_CRP POCT_Protocol Master Document. (PDF)

S1 Checklist CONSORT-extension-Pilot-and-Feasibility-Trials-Checklist https://doi.org/10.1136/bmj.i5239. (DOC)
