# Supplementary material for: Serial C-Reactive Protein Point-of-Care testing to optimize antibiotic treatment in hospitalized children with signs of infection in Zanzibar: A feasibility study
Source: PLOS Glob Public Health. 2025 Dec 23;5(12):e0004777. doi: 10.1371/journal.pgph.0004777 (PMC12725598; doi:10.1371/journal.pgph.0004777)
Supplement: S1 Text — (PDF) [file pgph.0004777.s001.pdf]

Participant ID								

HEALTHCARE WORKERS QUESTIONAIRE

PART A: Demographic Information

1. Gender
   a. Male
   b. Female

2. Age __/__/ years

PART B: Professional education and Experience

3. What is your current professional designation
   a. Doctor
   b. Pediatrician
   c. Nurse
   d. Pharmacist
4. How many years of professional experience do you have in your current role __/__ years
5. How many years have you been practicing in your current healthcare facility? __/__ years
6. What is your highest level of education?
   a. Bachelor’s degree
   b. Master’s degree
   c. Doctorate degree
7. Have you received any specific training or education on infectious diseases management?
   a. Yes
   b. No
8. If yes, please specify the type of training or education received.
   a) Workshop/seminar
   b) Conference
   c) Online courses

PART C: Acceptability of CRP Point-of-Care Testing

9. Are you familiar with C-reactive protein (CRP) point-of-care testing?
   a. Yes, very familiar
   b. Somewhat familiar
   c. Not familiar
10. Have you ever used CRP point-of-care testing in your clinical practice?
    a. Yes
    b. No
11. If yes, please indicate the context or setting in which you have used CRP point-of-care testing (e.g., outpatient clinic, emergency department, etc.)

Participant ID								

12. In your opinion, how important is it to have a rapid CRP point-of-care testing for guiding antibiotic treatment decisions in neonates and children with febrile illness and diarrhea?
    a. Very important
    b. Somewhat important
    c. Not important

13. How confident are you in interpreting CRP point-of-care test results?
    a. Very confident
    b. Somewhat confident
    c. Not confident

14. Do you think CRP point-of-care testing can help reduce unnecessary antibiotic prescribing in neonates and children with febrile illness and diarrhea?
    a. Yes, significantly
    b. Yes, to some extent
    c. No, not at all

Participant ID								

DODOSO KWA AJILI YA WAHUDUMU WA AFYA

SEHEMU A: Taarifa Binafsi

1. Jinsia
   a. Mwanaume
   b. Mwanamke

2. Umri: miaka __/____/

SEHEMU B: Elimu na uzoefu wa kitaalamu

3. Jina lako la kitaalamu la sasa ni nani
   a. Daktari
   b. Daktari bingwa wa watoto
   c. Muuguzi
   d. Famasia

4. Una muda gani katika nafasi yako ya kitaaluma ya sasa: miaka ___/____
5. Una muda gani katika kituo chako cha afya cha sasa miezi ___/___
6. Ngazi yako ya elimu ya juu zaidi?
   a. Shahada ya kwanza
   b. Shahada ya uzamili
   c. Shahada ya uzamivu
7. Je, umepata mafunzo au elimu maalum juu ya usimamizi wa magonjwa ya nayosababishwa na bakiteria?
   a. Ndiyo
   b. Hapana
8. Kama ndiyo, tafadhali eleza aina ya mafunzo au elimu uliopata
   a) Warsha
   b) Mkutano
   c) Kozi za mtandaoni

____

HAPA C: Kukubalika kwa upimaji wa protini ya "C-reactive" kwenye Sehemu ya huduma

9. Je, unaufahamu upimaji wa protini ya "C-reactive" kwenye Sehemu ya huduma?
   a. Ndiyo, ninajua vizuri sana
   b. Ninajua kidogo tu
   c. Sifahamu kabisa

10. Je, umewahi kutumia vipimo vya protini ya "C-reactive" kwenye sehemu ya huduma katika utendaji kazi wa kila siku hospitalini?
    a. Ndiyo

Participant ID								

b. Hapana

11. Kama ndiyo, tafadhali eleza mazingira au eneo ambalo umetumia vipimo vya vipimo vya protini ya “C-reactive” kwenye sehemu ya huduma (kwa mfano, kliniki ya nje, idara ya dharura, nk.)

____

12. Kwa maoni yako, ni muhimu kwa kiwango gani vipimo vya protini ya “C-reactive” kwenye sehemu ya huduma kusaidia katika kufanya maamuzi ya matibabu ya antibayotiki kwa watoto wachanga na watoto wenye homa na kuhara?
    a. Muhimu Sana
    b. Muhimu
    c. Muhim Kidogo
    d. Sio muhimu

13. Jinsi gani unajiamini katika kusoma matokeo ya vipimo vya protini ya “C-reactive” kwenye sehemu ya huduma?
    a. Nina jiamini sana
    b. Nina jiamini kidogo tu
    c. Sijiamini

14. Je, unafikiri vipimo vya protini ya “C-reactive” kwenye sehemu ya huduma vinaweza kusaidia kupunguza matumizi ya antibayotiki yasiyo ya lazima kwa watoto wachanga na watoto wenye homa na kuhara?
    a. Ndiyo, kwa kiasi kikubwa
    b. Ndiyo, kwa kiwango fulani
    c. Hapana, kabisa
