# Supplementary material for: Serial C-Reactive Protein Point-of-Care testing to optimize antibiotic treatment in hospitalized children with signs of infection in Zanzibar: A feasibility study
Source: PLOS Glob Public Health. 2025 Dec 23;5(12):e0004777. doi: 10.1371/journal.pgph.0004777 (PMC12725598; doi:10.1371/journal.pgph.0004777)
Supplement: S2 Text — (PDF) [file pgph.0004777.s002.pdf]

Effectiveness of CRP-POCT for optimising antibiotic treatment in neonates and children

Introduction to sub-study 2 of the Project.

In this study we intend to investigate if the CRP POCT can be used to screen for viral or bacterial infection, to support appropriate use of antibiotics and become a key in fighting against AMR in low- and middle-income countries.

Antimicrobial resistance (AMR) is estimated to be associated with nearly 5 million deaths, with 1 in 5 deaths occurring in children (1). Globally, bacterial pathogens are increasingly becoming resistant to antibiotics, constituting a significant threat to global health. AMR is more pronounced in children and neonates than in the general population, largely due to the overuse and misuse of antibiotics in these age groups (2). The indiscriminate use of antibiotics in children, both in the health care settings and through over the counter sales in community pharmacies and other medicine outlets, has also fueled the development of resistant strains (3), especially in resource limited settings like Tanzania (4). Combatting AMR in children requires comprehensive strategies that involve among other things; improving health care infrastructures, including diagnostic tests to guide specific antimicrobial therapies (5), regulating antibiotics use (6) and implementing proper infection prevention and control measures (7).

Current practice for diagnosing and testing for suspected bacterial infections in Tanzania, Zanzibar specifically, is mainly full blood picture (8), in contrast to the gold standard test which is blood culture and antimicrobial susceptibility testing (AST) (9). Globally, inflammatory markers are potential alternatives for diagnosis of suspected bacterial infections amongst which are interleukin tests, procalcitonin and C-reactive protein (CRP) testing (10). Testing interleukin and/or procalcitonin is relatively expensive to carry out on a routine basis and it requires sophisticated infrastructures (11). C-reactive protein presents a unique opportunity for use in routine clinical practices as it is relatively cheaper to perform and sustain especially when it is used as a point-of-care (POC) test at the vicinity of attending clinicians' rooms (12,13). CRP testing is already optimized in Tanzania, though it has not yet been fully integrated as a routine diagnostic tool across healthcare settings, and especially in Zanzibar (14–16).

C-reactive protein is an inflammatory biomarker in blood which can serve as a proper diagnostic test to quickly ascertain and establish the likelihood of a bacterial infection. Through POC testing of CRP, health care providers can make more accurate and targeted decisions regarding antibiotic prescription as they wait for a 3-day final culture and AST results (17). However, CRP alone lacks absolute specificity in distinguishing bacterial from non-bacterial infections, as elevated levels can also be observed in non-bacterial conditions such as viral infections, autoimmune diseases, and

ZAN-TOTS Sub-Study 2: Master Protocol

other inflammatory states. To improve diagnostic accuracy and antibiotic stewardship, an integrated approach combining serial CRP measurements with microbiological confirmation is increasingly being advocated (18).

We will assess the effectiveness of using the CPR POCT assay for optimizing antibiotic therapy 1) in neonates with or at risk of early onset sepsis (EOS) and 2) neonates with clinical signs of late onset sepsis (LOS) as well as 3) admitted children with febrile illness and diarrhoea.

Objectives

The aim of this study is to determine the feasibility and effectiveness of C-Reactive Protein Point of Care Test in clinical decision making on antibiotic treatment in neonates and children with febrile illness and diarrhoea admitted in Vitongoji Hospital and Mnazi Mmoja Hospital in Zanzibar.

Specific objectives

1. Feasibility
   a. To assess the feasibility of using CRP POCT in paediatric wards in selected hospital in a low-resource setting
2. Use of CRP in neonates
   a. To assess the safety and effectiveness of the CRP POCT at 18-24 hours of life guiding initiation of antibiotic therapy in neonates at risk of early onset sepsis
   b. To assess the safety and effectiveness of the CRP POCT in guiding discontinuation of antibiotic therapy in neonates (0-28 days) admitted with suspected sepsis
3. Use of CRP in children from 6 months to 12 years
   a. To assess the effectiveness of the CRP POCT in guiding discontinuation of antibiotic therapy in children 6 months to 12 years admitted with fever or diarrhoea
4. Etiology and resistance
   a. To establish the pathogens and resistance patterns in neonates and children with blood culture proven sepsis

ZAN-TOTO Sub-Study 2: Master Protocol

Hypothesis

The hypothesis is that using CRP POCT to support clinical decision on antibiotic treatment of neonatal and pediatric patients in Mnazi Mmoja and Vitongoji hospital will safely help to 1) reduce the use of prophylactic antibiotics in neonates with WHO risk factor of early onset sepsis and 2) ensure adequate and appropriate antibiotic treatment for neonates with sepsis. Furthermore, for children 6 months to 12 years, it is the hypothesis that 3) the use of CRP POCT will help to identify

the majority of children who suffer from non-bacterial infections and assist in withholding or discontinue antibiotic treatment when no benefit can be expected thus lowering unnecessary-use of antibiotics or shorten the duration of use.

Methodology

Study design

This study is a prospective open-label, individual randomised controlled clinical trial with 28 days blinded follow-up conducted in Vitongoji (VT) hospital and Mnazi Mmoja Hospital (MMH) in Zanzibar. Neonates at risk of early onset sepsis, admitted neonates (0-28 days) with clinical signs of sepsis and admitted children (6 months to 12 years) with febrile illness or diarrhoea that are eligible for participation and parents will be asked for informed consent. If informed consent is obtained the child will undergo randomization to intervention: CRP POCT measurement at 18-24 hours of life (at risk of early onset sepsis neonates) or CRP POCT at admittance and after 18-24 hours of treatment. Further treatment and discontinuation of antibiotics will be guided by flowcharts supporting the clinicians decision of treatment, please refer to description of intervention for further details. Participants will be randomly assigned (1:1) to either CRP-guided or standard-of-care antibiotic continuation/discontinuation. Both groups will be followed up by telephone up to 28 days after intervention. The principal investigator will not be aware of the results of the randomisation and follow-up will be blinded as well. CRP-level and treatment data will be recorded and entered in a central database.

ZAN-TDD Sub-Study 2: Master Protocol

Study setting

The project is conducted in Zanzibar, part of the United Republic of Tanzania which has a total population of 1,889,773, with females accounting for 974,281 (52%) and children under 18 years of age accounting for 826,848 (48%) of the population. Zanzibar is composed of two main islands: Unguja and Pemba. The two islands are separated by a channel approximately 50 kilometres (about 31 miles) wide. The healthcare infrastructure comprises over 300 health facilities, including 189 public health facilities, which serve as the backbone of accessible healthcare, and 135 private health facilities (19, 20).

The proposed trial will involve Mnazi Mmoja Hospital (MMH) and Vitongoji (VT) Hospital, which serve catchment populations of 1,346,322 and 543,441, respectively.

The MMH is the only public tertiary hospital in Zanzibar and is located in Unguja Island. It has 570 bed capacity, of which 124 (21.8%) are paediatric beds. There are three units: neonatal wards with 49 beds; non-neonatal children up to 3 years and has 36 beds; and children ≥3 – 12 years with 36 beds. There are five paediatricians, seven medical doctors, one clinical pharmacist and 44 nurses

(of which 24 nurses are in the neonatal unit). The total number of admissions in 2021 in the paediatric wards was 17800. MMH has laboratory infrastructure for diagnostics services including culture and AST. Laboratory data from May to October (six months) showed a total of 11,395 complete blood counts samples, and 429 blood culture samples were processed.

The VT Hospital is a public district (secondary) hospital located in Pemba Island and is the highest-level care facility on the island. It has a 150-bed capacity and 229 staff of various cadres. Currently only 42 beds are operational due to on-going renovation (of these 10 are paediatric beds). In the paediatric ward, VT has 5 medical doctors, and 2 nurses work in paediatric ward. The total number of admissions in 2021 in the paediatric wards was 818. The laboratory data at VT hospital from May to October 2021 (six months) showed a total of 44 blood samples were received and processed for complete Blood Counts (CBC).

The Public Health Laboratory-Ivo de Carneri (PHL-IdC), (http://www.phlidc.org/), an entity of Ministry of Health located in Pemba, Zanzibar, United Republic of Tanzania, is a WHO Collaborating Centre for Neglected Tropical Diseases since 2005. Its activities include assisting relevant programmes to implement disease control strategies, undertaking operational research and strengthening capacities of health staff. PHL-IdC also provides the local population with routine services for selected laboratory tests to compliment Government MoH diagnostic and preventive efforts. It is the only public health laboratory in Pemba capable to carry out the microbiological diagnosis. It supports VT hospital and other hospitals in Pemba for culture and AST.

The microbiology laboratory at Makunduchi Health Centre, established in partnership with the Bernhard-Nocht Institute for Tropical Medicine (BNITM) provides diagnostic infrastructure for the study in Unguja island. This clinical laboratory in Zanzibar provides culture and AST and plays an important role in AMR Surveillance.

ZAN-TOTO Sub-Study 2: Master Protocol

Study population

Population

All neonates at risk of/with early onset sepsis, admitted neonates (0-28 days) with suspected sepsis and admitted children (6 months to 12 years) with febrile illness or diarrhoea in VT hospital and MMH are eligible for participation.

Inclusion criteria

Patients meeting the following criteria will be included in the study:

- Between 0-28 days (inclusive) and fulfilling one of two inclusion criteria

- Newborns at risk of early onset sepsis according to the WHO criteria and therefore eligible for prophylactic antibiotic treatment example:
    a) membranes ruptured over 18 hours before delivery or
    b) maternal fever (over 38 °C) during labour or
    c) amniotic fluid is foul smelling or purulent
  - All children from day 0 to day 28 admitted with suspected sepsis (clinical signs subjectively judged by the clinician)
- Children between 6 months and 12 years with need of admission with either:
  a) Febrile illness with temperature above 38 degrees or below 36 degrees at admission, or a history of febrile illness within the last 72 hours
  b) Diarrhoeal disease (defined as the passage of 3 or more loose or liquid stools per day) with or without fever
- Parents/caregivers of admitted children are able and willing to comply with all study requirements.
- Parents/caregivers of admitted children are able and willing to give voluntary Informed consent.

Exclusion criteria

- Age >28 days until 6 months
- Severely ill where measurement of CRP POCT would delay the treatment process.
- Neonates who need surgery or have major congenital malformations that will required hospital admission
- Patients with known immunosuppression or severe chronic disease
- Parents/caregivers who are not able to participate in follow-up procedures
- Positive rapid diagnostic test for malaria
- Have taken antibiotics within 24 hours before admittance

ZAN-TOTI Sub-Study 2: Master Protocol

Intervention

CRP POCT equipment will be supplied at the two hospitals MMH and VT hospital, along with a training in use and interpretation supporting the clinical evaluation of the neonate and child.

a) All included neonates delivered at one of the two hospitals with a risk factor of early onset sepsis as defined by WHO will have a CRP POCT measured at 18-24 hours of life. A measurement below 30mg/L indicates that the neonate does not need antibiotic treatment, CRP levels between 30mg/L and 50mg/L indicate caution and that the neonate should be

assessed clinically and CRP measurement repeated after 6 hours. CRP levels above 50mg/L indicate that the neonate needs antibiotic treatment for early onset sepsis, figure 1.

b) For neonates admitted with suspected sepsis and randomized to the CRP group, antibiotic treatment should be initiated regardless of CRP levels and if possible including a blood culture. CRP will be reassessed after 18-24 hours of treatment, if both CRPs are below 10mg/L antibiotics are most likely not needed. The treatment is continued until the result of the blood culture, if negative the clinician can consider differential diagnosis and discontinuing the antibiotic treatment if the neonate is well. CRP levels above 100mg/L indicates severe disease and that the clinician should take into consideration meningitis and performing a lumbar puncture, figure 2.

c) For children 6 months to 12 years admitted with fever, history of fever or diarrhoea and in the intervention group, CRP will be taken at admittance and again at 18-24 hours. It will be communicated that if both CRP levels are less than 10mg/L it indicates that the disease is not severe, and antibiotics are most likely not needed and hence can be either not started or discontinued. If the second CRP is between 10mg/L and 50mg/L, antibiotics might be needed depending on whether the trend of the CRP is upwards or downwards. Hence, if antibiotics have been initiated the clinician can consider stopping the antibiotics earlier. If any of the CRP's are more than 50mg/L that it is likely that antibiotics are needed and treatment should be continued, figure 3. At all times the health care workers are instructed to take the clinical picture into account together with the value of the test. With obvious symptoms of bacterial infection with affected general condition, antibiotic treatment may be initiated and continued on clinical indication. The training of health care workers will also include knowledge of CRP pharmacodynamics and cases where a low CRP might need to be interpreted cautiously, e.g. a history of fever lasting less than 24 hrs. HCWs will be instructed to use CRP POCT for all patients in the intervention group and use the information to guide diagnosis and treatment choice. We will use an appropriate machine procured through the Ministry of Health, for instance Aidian QuickRead go CRP POCT set-up. Feasibility of using the CRP POCT will be assessed using the framework provided by Bowen et al describing acceptability, demand, implementation, practicality, adaptation, integration, expansion, limited-efficacy testing (4).

ZAN-TIIO Sub-Study 2. Master Protocol

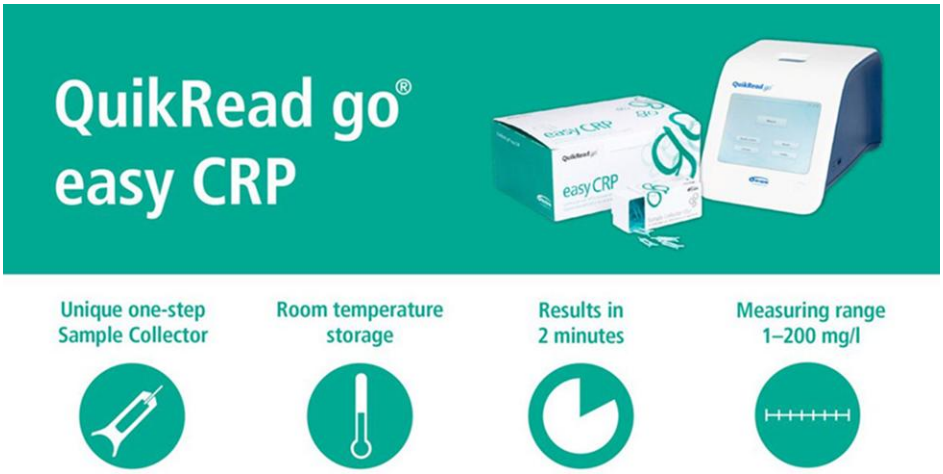

Aidian CRP POCT.

The training of HCWs will include the following flowcharts, figure 1 to 3

Protocol

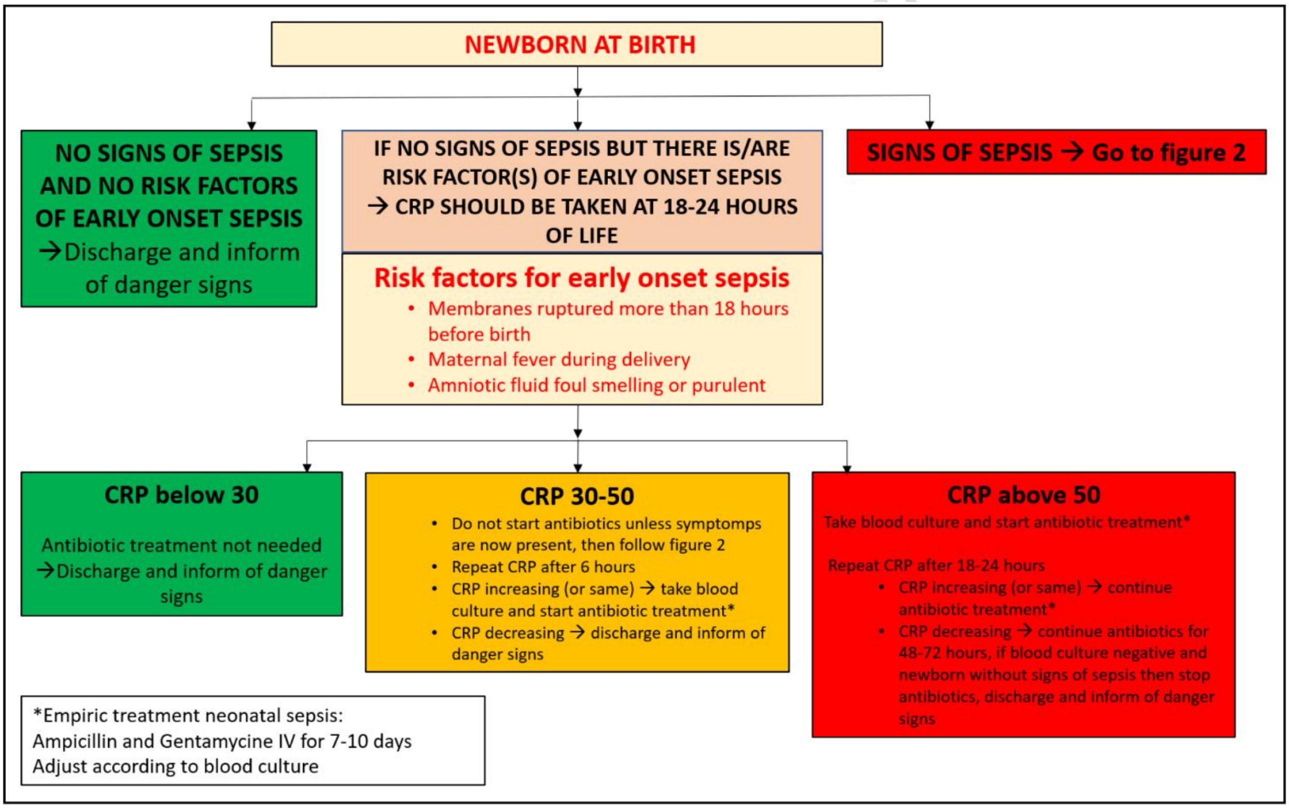

Figure 1: CRP intervention flowchart for neonates with risk factors of early onset sepsis (EOS)

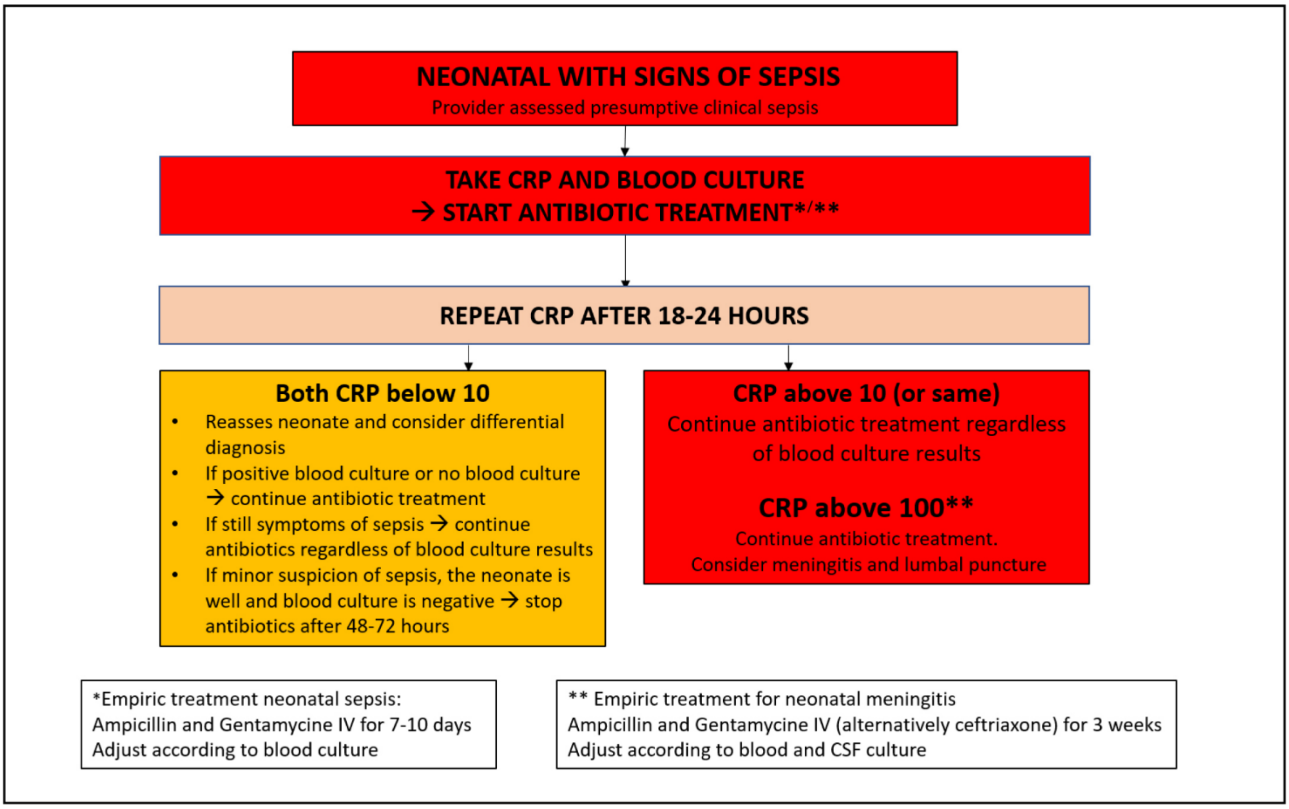

Figure 2: CRP intervention flowchart for neonates with signs of infection

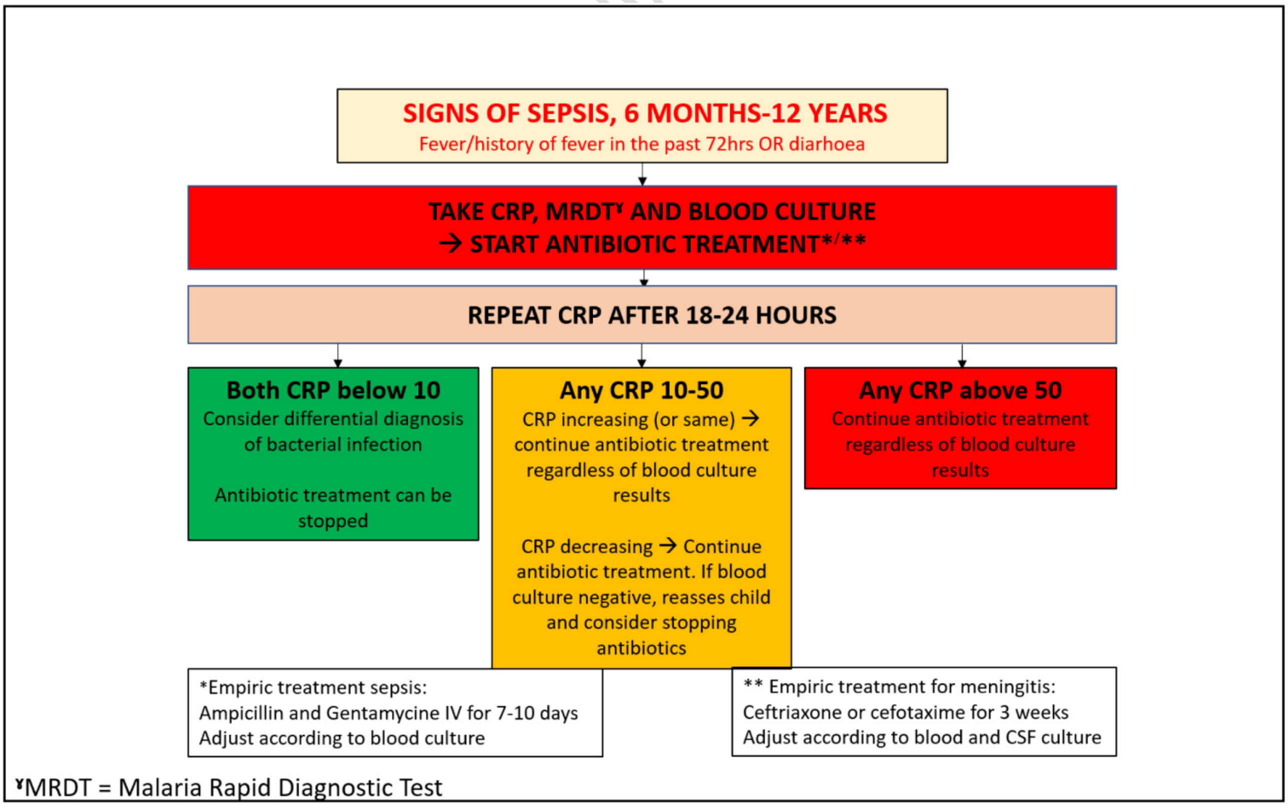

Figure 3: CRP intervention flowchart for children with febrile illness or diarrhea

Study endpoints

The primary study outcome is the duration of antibiotic treatment (number in 24-hour periods between start and end of antibiotic treatment) within 14 days of the admittance of included neonates and children in each study arm (superiority analysis). Antibiotic use during admittance and a composite measure of antibiotic use during admittance and discharge are reported. For every participant, the types and quantity of antibiotics given during the study period will be assessed based on individual drug administration records. To ensure patient safety in case of reduction in antibiotic use a second primary outcome relapse of infection is measured as reinstitution of antibiotics within 72 h after completion of the initial course of antibiotic treatment.

The secondary study outcomes are:

1. Antibiotic use (prescribed daily doses)
2. Choice of antibiotic drug and dose compared to local standard treatment guidelines
3. Number of days admitted in hospital
4. Number of days until resolution of symptoms
5. Development of clinical signs of sepsis
6. Proportion of cases with blood cultures collected before antibiotic treatment
7. Bacterial pathogens and AST in blood cultures
8. Hospital re-admittance within 14 days
9. Mortality at follow up day 7 and 28

Our definition of prescribed and defined daily doses accords with the recommendations of WHO. The route of administration will be incorporated in the daily dose calculations.

Sample size calculation

For the superiority primary outcome, the sample size calculation is based on an estimated 20% reduction in duration of antibiotic treatment. Assuming the mean duration of antibiotic treatment of 7 days (168 hours) and an SD of 5 days (120 hours), with an α of 0.05 and a β of 0.1. The trial needs 262 patients in each group (524 patients in total). However, some patients may be discharged before reaching the stopping rules and these patients would not be stopped according to the CRP guidelines. We assume that 20% of patients are going to be discharged before the stopping rule was enacted or lost to follow-up. To ensure enough power for independent analysis for the three study population groups we apply this power calculation for each group. Furthermore, for children 6 months to 12 years further inflation of sample size by 50% is done to ensure exploration of sub-groups with different symptoms of infection (e.g. pneumonia, diarrhoea etc.). To establish non-inferiority of the second primary outcome of relapse of infection, assuming event rate

ZAN-TIDD Sub-Study 2: Master Protocol

of 5% of relapse, non-inferiority margin of 6%, one-sided alpha of 2.5%, power of 90% the sample size is 278 per group i.e. total 556 children plus 20% loss to follow-up equaling 646 children.

Hence, the study aims to include 646 neonates at risk of infection, 646 neonates with suspected infection and 969 children aged 6 months to 12 years with infection - an overall total of 2261 children included. Power calculations were performed using sample size calculators provided by Sealed Envelope (21).

Randomisation and blinding

We plan not to use the cluster randomization because it is not possible in the Zanzibar setting due to the limited number of sites (two) while cluster randomization requires a large number of sites that are relatively comparable. Furthermore, the current study in Kirgizstan has shown that individual randomization is feasible and can be applicable in Zanzibar (22). The randomisation will be carried out by a responsible Research Assistant through the Sealed Envelop™ programme (www.sealedenvelope.com). This person will inform the HCW directly of the result of the randomization. Follow-up will be blinded as well. Participants will be randomised (1:1) to either standard care or care according to CRP POCT. Randomisation will be stratified by male/female as well as within the three study groups.

Study procedures

Participants are screened for eligibility at the participating hospitals by a research assistant (RA) prior to routine examination by a clinician. Parents /caregivers of eligible children are requested for informed consent, after having received oral and written information regarding the trial. Participants are then randomized to the intervention group (CRP POCT) or control group (usual care) by a responsible RA through the REDCap Randomization Module (23). Case report forms (CRFs) are filled out by RA for all participants at the first consultation and every subsequent day the participant is admitted. Participants assigned to the intervention group are provided with CRP POCT during consultation with their clinician at admittance and after 18-24 hours of treatment. The CRP POCT is carried out as a prick test using capillary blood. Children in the control group do not have the CRP POC test taken. Decision on the treatment of participants is exclusively the responsibility of the clinicians of the included hospitals. To assess adherence, all treatment decisions including deviations from CRP recommendations are recorded in case report forms (CRFs). Reasons for deviation are documented for transparency and allow for subgroup analysis.

During admission RA follow the enrolled children daily and register

- Demographic details including sex, age, co-morbidities
- Obstetric and neonatal background information
- Duration of clinical symptoms prior to consulting
- Presence of specific and general symptoms
- Clinical assessments and results of clinical examination (oxygen saturation, respiratory rate, blood pressure, heart rate, body temperature)
- All diagnostic tests performed or ordered
- Management: antibiotic, antiviral prescription, other (symptomatic) medication prescribed and advised
- Additional advice: preventive measures for themselves and their family members, time-off, home isolation, hygienic measures
- Referral to other hospitals

Furthermore, HCWs in participating hospitals answer a questionnaire on professional education as well as acceptability of CRP POCT testing during the feasibility study.

Follow-up by phone

After discharge from the hospital, all participants are contacted on the 7th and 28th day by phone calls from a RA who is blinded to the group allocation. Information gathered on the follow-up call includes general medical history of the child post discharge, additional diagnostic testing (with pictures of medical records or clinical follow-up if possible), complications (such as hospitalization, days in hospital, ICU stay, medication/oxygen/ventilation received, pneumonia, or death), and any medicine bought over-the-counter or prescribed by other healthcare workers (including the name of the drug, route of administration, frequency of administration and dose).

Training of HCW

Before initiation of the study all HCW in the relevant departments in MMH and CHH will receive an in-house training on CRP POCT. The training will include how to use CRP POCT and interpretation supporting the clinical evaluation of the child. The training will be performed by the PI and research team.

Eligibility Assessment

Neonates 0-28 days and children 29 days to 12 years in the two hospitals MMH and CHH will be assessed against the inclusion criteria by a research assistant. The research assistant will be present in the wards 24 hours during the days Monday to Friday.

Informed Consent

Parents or caregivers of children eligible for inclusion will be approached by research assistants and explained about the study orally and in writing if able to read. The information will include the exact nature of the study, implications and constraints as well as risks and benefits of participation. It will be clearly stated in the patient information sheet and verbally explained to the participant that they are free to withdraw from the study at any time for any reason without impacting their future care, and with no obligation to provide the reason for withdrawal. Consent will be approved by signature or fingerprint by one of the parents or legal caregiver as the participating children are not of legal age.

Data Management and analysis

Data management

Data collection and management will be overseen by the PhD student. In addition each hospital will have a supervisor/research coordinator responsible for data collection and management in that hospital. Data will be entered directly into REDCap through secure tablets and transferred to STATA where analysis will be performed. Informed consent forms will be on paper and all data will be kept confidentially.

Data collection tools

The data collection materials will consist of:

- Informed consent (English and Swahili), appendix 2.1, will be asked by the research assistants.
- Description of available drugs, equipment for treatment such as peripheral venous catheters, i.v. fluids. This will be performed two times during the study, at initiation and end of data collection. The data will be collected by a research assistant. The content for available material is shown in appendix 2.2.
- Inclusion and eligibly assessment for neonates and children respectively, appendix 2.3 and 2.4. The risk factors according to the WHO's guidelines for neonatal sepsis and the use of prophylactic antibiotic will be assessed using a picture of the partogram, the delivery book, the antenatal card and the case file. The admitted neonates and children eligible assessment will be done by case files and admittance books.

ZAN TOTO Sub-Study 2. Master Protocol

- After inclusion the caregiver assisted by the research assistant will complete a questionnaire (English and Swahili) on socio-economic background and health seeking behaviour, appendix 2.5.
- CRP will be taken by the clinician responsible for treatment supported by the research assistant responsible for randomisation and data collection. Clinical data and treatment registration will also include the general examination, respiratory count, temperature, and a blood glucose measurement (capillary point-of-care test) and prescriptions. Emphasis will be put on the use of antibiotics during admission and status of child obtained from the hospital files, appendix 2.6.
- At the time of discharge or death a discharge form will be filled, appendix 2.7. The family will be contacted to enquire about the status of the child after discharge at day 7 and 28 primary by phone, but if not available by home visit of the research assistant, appendix 2.8.
- Questionnaire for the health care worker on their education, work experience and acceptability of using CRP will be conducted at the feasibility phase of the study using a semi-structured questionnaire, appendix 2.9.

Appendix 2.1: Informed consent
Appendix 2.11: Informed consent for healthcare personnel
Appendix 2.2: Available material survey
Appendix 2.3: Inclusion and randomization neonates
Appendix 2.4: Inclusion and randomization children
Appendix 2.5: Socioeconomic background and health seeking behavior
Appendix 2.6: Clinical data registry
Appendix 2.7: Discharge form
Appendix 2.8: Follow-up form
Appendix 2.9: Health worker education and acceptability form

ZAN-TOTO Sub-Study 2: Master Protocol

Data analysis

Data analysis will include comparing baseline variables between the intervention and control groups using X2 tests for categorical variables and t-tests for continuous variables, without adjustment. Baseline characteristics such as age, sex, urban/rural residence, number of children, and study site will be compared. Separate analyses will be conducted for all three sub-groups neonates at risk of infection, neonates with signs of infection and children aged 6 months to 12 years.

The primary outcome duration of antibiotic treatment (days), which is a continuous variable, will be compared between the intervention and control groups using a multivariable linear regression model. Neonates at risk of early onset sepsis are recommended for two days of antibiotic treatment by the WHO. Therefore, in this subgroup, an additional non-inferiority analysis will be conducted using a multivariable logistic regression model, comparing CRP POCT with prophylactic two-day antibiotic treatment on the binary outcome of antibiotic prescribed yes/no. All comparisons will be conducted using both unadjusted and adjusted models for sex, age, and clinical status, following an intention-to-treat (ITT) approach. To account for deviations from CRP-based recommendations, a secondary Per-Protocol (PP) analysis will be conducted, excluding participants who did not adhere to their assigned intervention. For neonates at risk of early-onset sepsis, a non-inferiority comparison will be performed per protocol, assessing whether CRP-guided management is not inferior to standard clinical decision-making. Adherence to CRP-based recommendations will be systematically recorded and analyzed to evaluate its impact on treatment decisions. Statistical analyses will be performed after entering data from the final follow-up and data will be stored in a REDCap database for analysis using STATA.

Safety reporting

Data Safety Monitoring Board (DSMB)

A Data Safety Monitoring Board (DSMB) has been established to conduct interim analyses, including the evaluation of SAEs, which will be presented and discussed. The primary duties of the DSMB include regularly reviewing and assessing the accumulated study data for participant safety, study progress, and, when applicable, efficacy, and providing recommendations to the Principal Investigator regarding the continuation, modification, or termination of the trial. The DSMB is considering both study-specific data and relevant background information about the disease, test agent, or patient population under investigation. Members of the DSMB are independent clinicians and researchers from Denmark and Tanzania not directly involved in the current study.

Quality Assurance

Quality assurance of the study will include:

PhD student: A PhD student will be employed to oversee the study and as a principal investigator. The PhD student will be enrolled at a regional university and will be supervised by senior researchers from Global Health Unit (GHU) and the Catholic University of Health and Allied Sciences (CUHAS).

ZAN-TRIO Sub-Study 2: Master Protocol

Master students: Two master students will be employed to assist the phD student and participate in the overall project team. The master students will be enrolled at a regional university in a master programme suitable for the project and of their own interest.

Training of Research Assistants: Research Assistants will be selected at all included facilities. They will receive a one-day course on data collection. On site supervision and data control will be performed by the supervisor

Monitoring: The PhD student and site supervisors will monitor data collection and provide support on a daily basis.

Supervision: The international research team will oversee the data collection and PhD student. Site visits will be performed at least two times during the data collection.

DSMB will oversee the safety of the study, and the project management structure will ensure the implementation and coordination of the three sub-studies.

ZAN-TOTO Sub-Study 2: Master Protocol

References

1. Murray CJL, Ikuta KS, Sharara F, Swetschinski L, Aguilar GR, Gray A, et al. Global burden of bacterial antimicrobial resistance in 2019: a systematic analysis. Lancet. 2022;399(10325):629–55.
2. Romandini A, Pani A, Schenardi PA, Pattarino GAC, De Giacomo C, Scaglione F. Antibiotic resistance in pediatric infections: global emerging threats, predicting the near future. Antibiotics. 2021;10(4):393.
3. Laxminarayan R, Duse A, Wattal C, Zaidi AKM, Wertheim HFL, Sumpradit N, et al. Antibiotic resistance—the need for global solutions. Lancet Infect Dis. 2013;13(12):1057–98.
4. Seni J, Mapunjo SG, Wittenauer R, Valimba R, Stergachis A, Werth BJ, et al. Antimicrobial use across six referral hospitals in Tanzania: A point prevalence survey. BMJ Open. 2020;10(12):1–9.
5. Frost I, Laxminarayan R, McKenna N, Chai S, Joshi J. Antimicrobial resistance and primary health care. World Health Organization. World Health Organization; 2018. Available from: https://apps.who.int/iris/bitstream/handle/10665/326454/WHO-HIS-SDS-2018.56-eng.pdf%0Ahttps://www.who.int/docs/default-source/primary-health-care-conference/amr.pdf?sfvrsn=8817d5ba_2
6. da Silva ARA, de Almeida Dias DCA, Marques AF, di Biase CB, Murni IK, Dramowski A, et al. Role of antimicrobial stewardship programmes in children: a systematic review. J Hosp Infect. 2018;99(2):117–23.
7. Cotton MF, Sharland M. Antimicrobial stewardship and infection prevention and control in low-and middle-income countries: current status and best practices. Pediatr Infect Dis J. 2022;41(3S):S1–2.
8. Iregbu K, Dramowski A, Milton R, Nsutebu E, Howie SRC, Chakraborty M, et al. Global health systems' data science approach for precision diagnosis of sepsis in early life. Lancet Infect Dis. 2022;22(5):e143–52. Available from: http://dx.doi.org/10.1016/S1473-3099(21)00645-9
9. Leong K, Gaglani B, Khanna AK, McCurdy MT. Novel diagnostics and therapeutics in sepsis. Biomedicines. 2021;9(3):1–23.
10. Barichello T, Generoso JS, Singer M, Dal-Pizzol F. Biomarkers for sepsis: more than just fever and leukocytosis—a narrative review. Crit Care. 2022;26(1):14.
11. Li CH, Seak CJ, Chaou CH, Su T -H, Gao SY, Chien CY, et al. Comparison of the diagnostic accuracy of monocyte distribution width and procalcitonin in sepsis cases in the emergency department: a prospective cohort study. BMC Infect Dis. 2022;22(1):1–10. Available from: https://doi.org/10.1186/s12879-021-06999-4

12. Oppong R, Jit M, Smith RD, Butler CC, Melbye H, Mölstad S, et al. Cost-effectiveness of point-of-care C-reactive protein testing to inform antibiotic prescribing decisions. Br J Gen Pract. 2013;63(612):e465–71.
13. Hunter R. Cost-Effectiveness of Point-of-Care C-Reactive Protein Tests for Respiratory Tract Infection in Primary Care in England. Adv Ther. 2015;32(1):69–85. Available from: https://doi.org/10.1007/s12325-015-0180-x
14. Chacha F, Mirambo MM, Mushi MF, Kayange N, Zuechner A, Kidenya BR, et al. Utility of qualitative C-reactive protein assay and white blood cells counts in the diagnosis of neonatal septicaemia at Bugando Medical Centre, Tanzania. BMC Pediatr. 2014;14:1–8.
15. Althaus T, Lubell Y, Maro VP, Mmbaga BT, Lwezaula B, Halleux C, et al. Sensitivity of C-reactive protein for the identification of patients with laboratory-confirmed bacterial infections in northern Tanzania. Trop Med Int Heal. 2020;25(3):291–300.
16. Hildenwall H, Muro F, Jansson J, Mtove G, Reyburn H, Amos B. Point-of-care assessment of C-reactive protein and white blood cell count to identify bacterial aetiologies in malaria-negative paediatric fevers in Tanzania. Trop Med Int Heal. 2017;22(3):286–93.
17. Althaus T, Greer RC, Swe MMM, Cohen J, Tun NN, Heaton J, et al. Effect of point-of-care C-reactive protein testing on antibiotic prescription in febrile patients attending primary care in Thailand and Myanmar: an open-label, randomised, controlled trial. Lancet Glob Heal. 2019;7(1):e119–31.
18. Gurumoorthy K. Serial quantification of CRP and total leukocyte count as a complementary tool in neonatal sepsis. Bioinformation. 2022;18(10):920–4.
19. United Republic of Tanzania(URT). Census Information Dissemination Platform. 2022 [cited 2023 June 10]. Available from: https://sensa.nbs.go.tz/
20. Ministry of Health Zanzibar. Zanzibar Annual Health Information Bulletin 2021. 2021; Available from: https://mohz.go.tz/eng/health-bulletins
21. Sealed Envelope. Sealed Envelope Ltd. 2020 [cited 2023 Jun 13]. Simple randomisation service | Sealed Envelope. Available from: https://sealedenvelope.com/simple-randomiser/v1
22. Isaeva E, Bloch J, Poulsen A, Kurtzhals J, Reventlow S, Siersma V, et al. C reactive protein-guided prescription of antibiotics for children under 12 years with respiratory symptoms in Kyrgyzstan: protocol for a randomised controlled clinical trial with 14 days follow-up. BMJ Open [Internet]. 2023 Apr 1;13(4):e066806. Available from: http://bmjopen.bmj.com/content/13/4/e066806.abstract
23. Kianersi S, Luetke M, Ludema C, Valenzuela A, Rosenberg M. Use of research electronic data capture (REDCap) in a COVID-19 randomized controlled trial: a practical example.

BMC Med Res Methodol. 2021;21(1):175. Available from: https://doi.org/10.1186/s12874-021-01362-2

ZAN-TOTO Sub-Study 2: Master Protocol
